# Supplementary material for: National-scale biogeography and function of river and stream bacterial biofilm communities
Source: Nat Commun. 2025 Nov 26;16:10571. doi: 10.1038/s41467-025-65620-3 (PMC12657883; doi:10.1038/s41467-025-65620-3)
Supplement: Supplementary file 1 — Supplementary Information [file 41467_2025_65620_MOESM1_ESM.pdf]

Supplementary Information

Supplementary Table 1. Number of sampling sites and the dominant land cover type and geology in the upstream catchment. Source data are provided as a Source Data file.

Dataset	Type	Number of sites
Land use	Acid grassland	24
	Arable and horticulture	47
	Bog	5
	Broadleaved woodland	2
	Coniferous woodland	2
	Heather	5
	Heather grassland	4
	Improved grassland	54
	Neutral grassland	1
	Suburban	2
Geology	Calcareous	77
	Chalk	19
	Peat	7
	Siliceous	43

Supplementary Table 2. Summary of water chemistry data including water temperature (°C), pH, alkalinity to pH 4.5 as $CaCO_3$ (mg L^{-1}), conductivity, and the concentration of chloride ion (mg L^{-1}), dissolved oxygen (DO, mg L^{-1}), dissolved oxygen saturated (mg L^{-1}), dissolved organic carbon (DOC, mg L^{-1}), total phosphorus (TP, mg L^{-1}), orthophosphate (mg L^{-1}), total nitrogen (TN, mg L^{-1}), total oxidised nitrogen (TON, mg L^{-1}), nitrate-nitrogen (nitrate-N, mg L^{-1}), nitrite-nitrogen (nitrite-N, mg L^{-1}), ammoniacal nitrogen (ammoniacal-N, mg L^{-1}), ammonia-N unionised (mg L^{-1}), and reactive SiO_2 (mg L^{-1})**.** Source data are provided as a Source Data file.

	Mean	SD	SE	Min	Max	Median	Count
Temperature	11.02	4.13	0.20	2.30	19.97	11.50	444
pH	7.82	0.53	0.02	5.18	8.80	7.93	444
Alkalinity	152.73	86.74	4.31	5.00	446.67	163.33	405
Conductivity	521.13	553.03	26.25	24.00	7748.33	458.33	444
Chloride ion	47.99	128.08	6.38	4.03	2293.33	25.75	403
DO	10.70	1.87	0.09	2.55	14.70	10.88	444
DO saturated	96.09	12.29	0.58	25.03	145.63	97.80	444
DOC	4.93	4.13	0.20	0.33	41.50	3.75	443
TP	0.16	0.47	0.02	<0.01	8.10	0.08	400
Orthophosphate	0.11	0.35	0.02	0.00	6.20	0.04	443
TN	6.96	4.34	0.30	0.52	27.00	6.05	214
TON	4.08	4.03	0.19	0.01	20.67	2.80	443
Nitrate-N	4.06	4.02	0.19	<0.01	20.57	2.79	443
Nitrite-N	0.02	0.03	<0.01	<0.01	0.26	0.01	443
Ammoniacal-N	0.08	0.25	0.01	<0.01	4.57	0.04	443
Ammonia-N unionised	<0.01	0.03	<0.01	<0.01	0.68	<0.01	443
SiO_2	7.07	3.44	0.16	1.02	24.00	6.80	441

Mean, standard deviation (SD), standard error (SE), minimum, maximum, and median are calculated from the 3 months mean (mean of up to 5 measurements taken during a 3-month period prior to biofilm sampling) across up to 450 samples collected from 146 sites between 2021 and 2023. Count is the number of river biofilm samples with a paired water chemistry measurement.

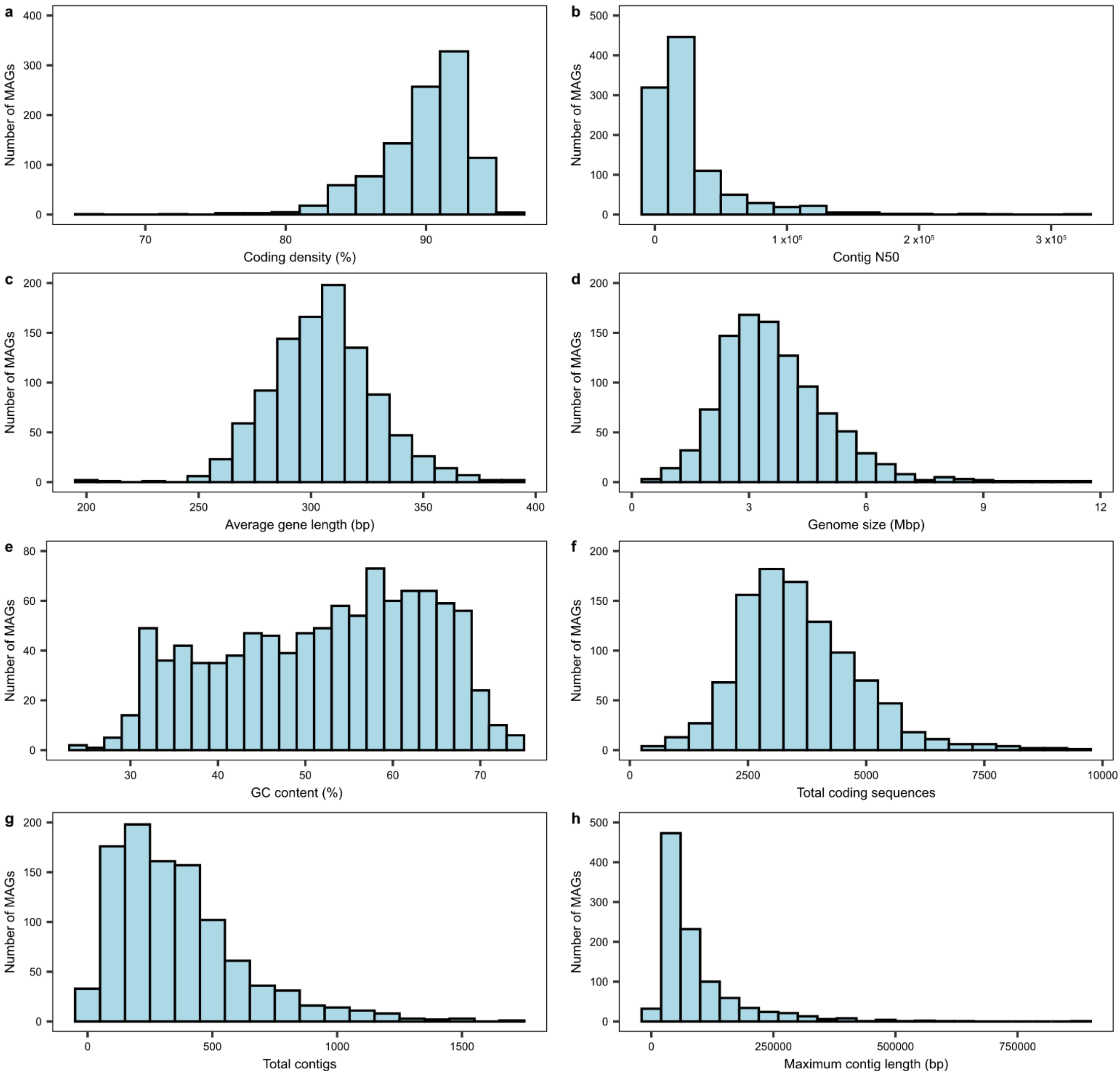

Supplementary Fig. 1. Distribution of MAG traits. (a) Coding density, (b) contig N50, (c) average gene length, (d) genome size, (e) GC content, (f) total coding sequences, (g) total contigs, and (h) maximum contig length. Source data are provided as a Source Data file.

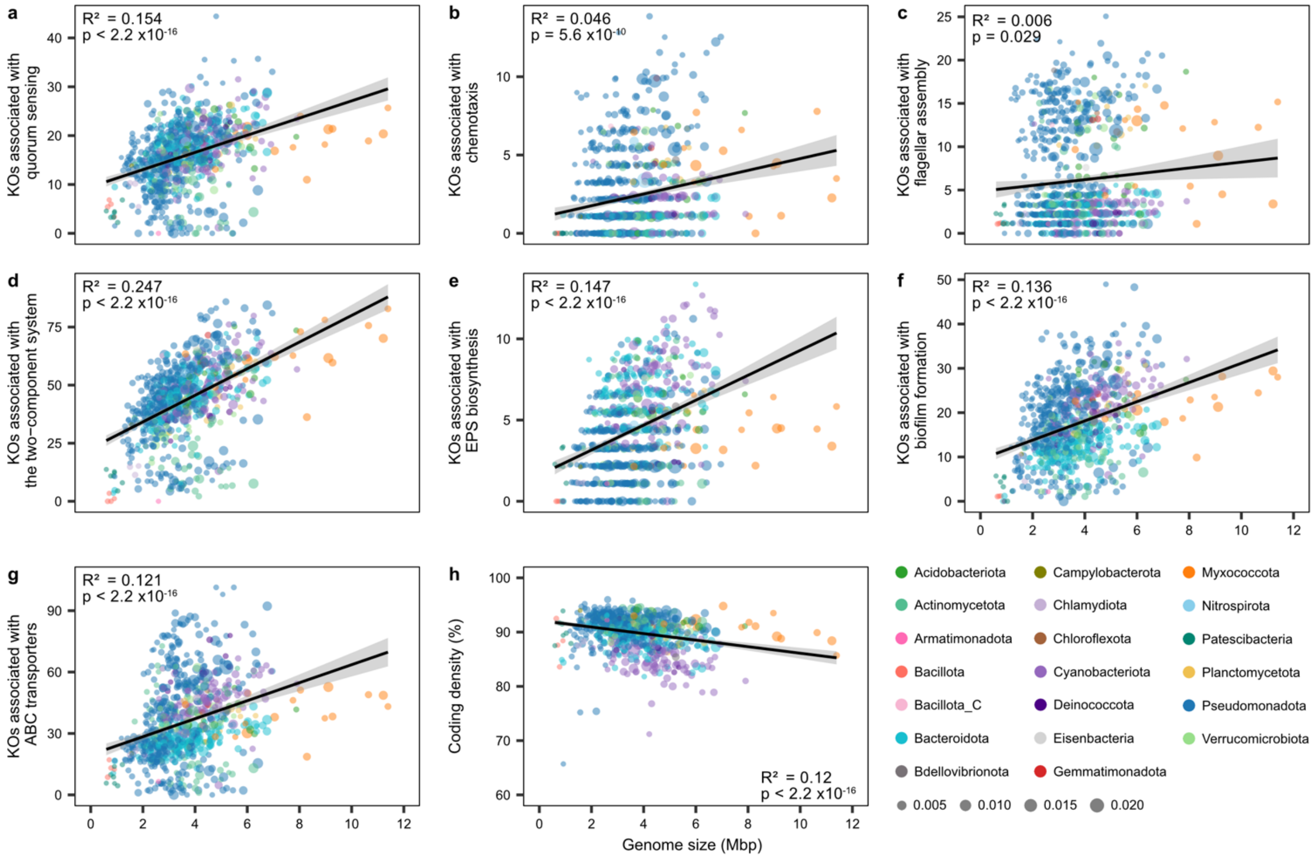

Supplementary Fig. 2. Biofilm associated genes identified in the bacterial MAGs. Genes associated with (a) quorum sensing, (b) chemotaxis, (c) flagellar assembly, (d) the two-component system, (e) exopolysaccharide (EPS) biosynthesis, (f) biofilm formation, and (g) ATP-binding cassette (ABC) transporters. The number of biofilm-associated KOs identified were normalised to (h) coding density for each MAG. Point size is scaled to mean relative abundance and colour represents bacterial phylum. Black lines and grey shading represent the linear regression with 95% confidence intervals and R^2 and significance levels are displayed. Source data are provided as a Source Data file.

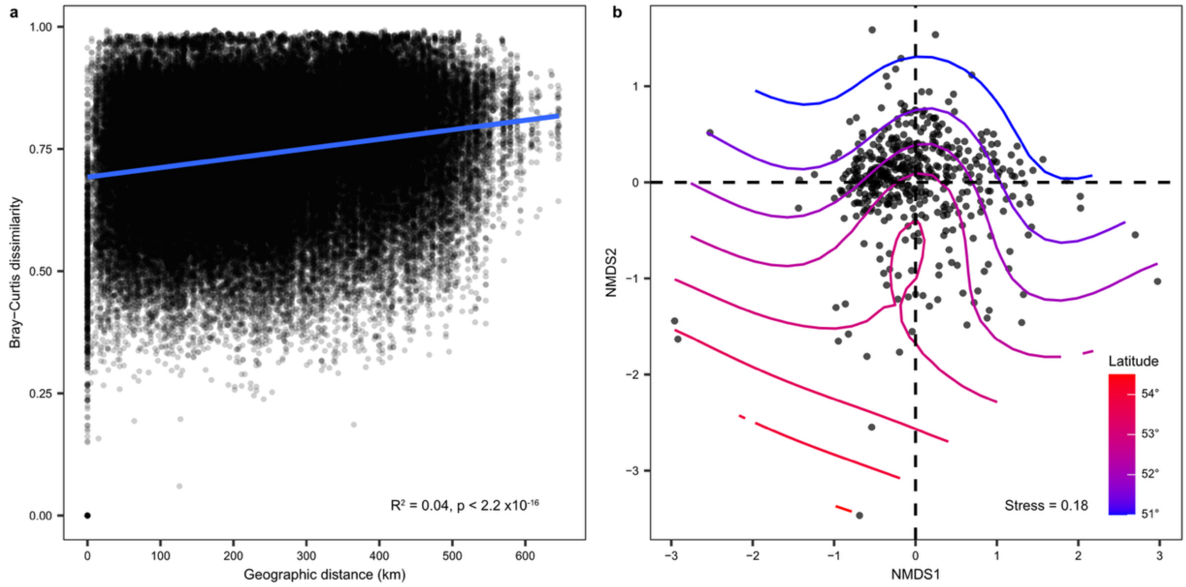

Supplementary Fig. 3. Distance-decay and beta-diversity of river biofilm bacterial communities. (a) Distance-decay relationship between Bray-Curtis community dissimilarity and geographic distance, R^2 and p values of the linear regression are shown. (b) Non-metric multidimensional scaling (NMDS) of a Bray-Curtis dissimilarity matrix based on beta diversity, latitude is represented by contour lines where the red to blue gradient indicate north to south. Source data are provided as a Source Data file.

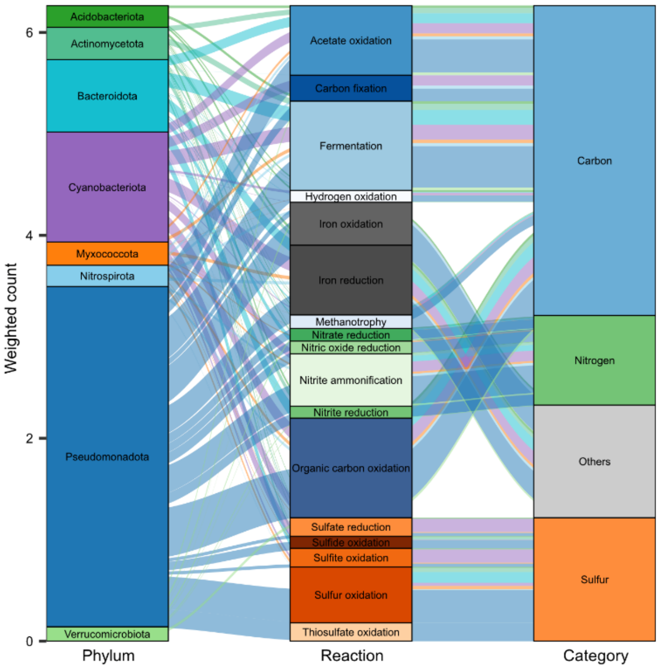

Supplementary Fig. 4. Alluvial plot of metabolic pathways identified in the MAGs. Weighted count is gene count weighted by mean relative abundance and summed by bacterial phylum. Only phyla and reactions with total weighted count >0.1 are shown for clarity. Source data are provided as a Source Data file.

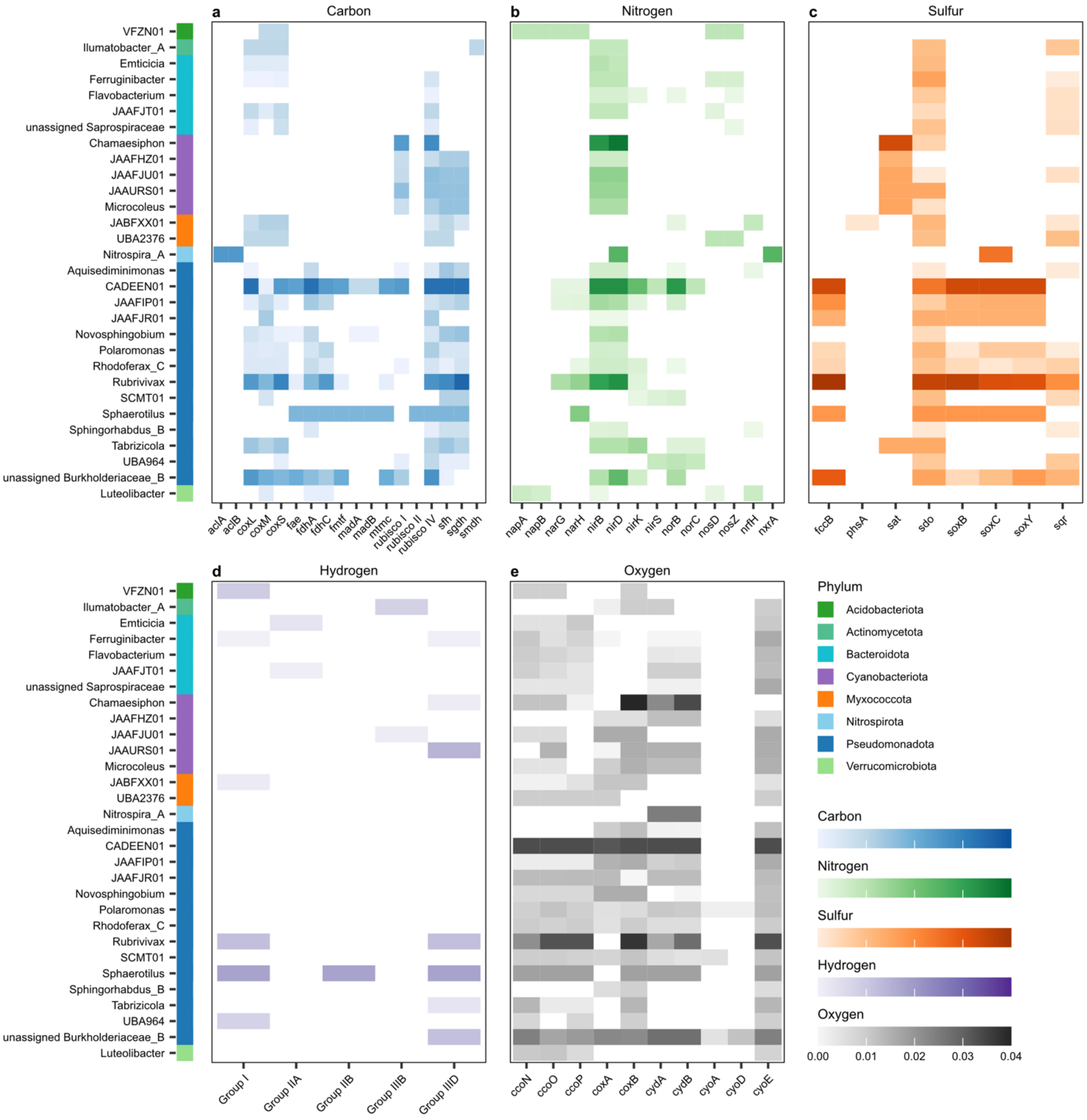

Supplementary Fig. 5. Nutrient cycling potential of bacterial MAGs presented at the genus level. Heatmaps of (a) carbon, (b) nitrogen, (c) sulfur, (d) hydrogen, and (e) oxygen cycling genes, where colour scales represent gene counts weighted by mean relative abundance and summed by bacterial genera. The 30 most abundant bacterial genera are shown, colours next to genera names represent bacterial phylum. Source data are provided as a Source Data file.

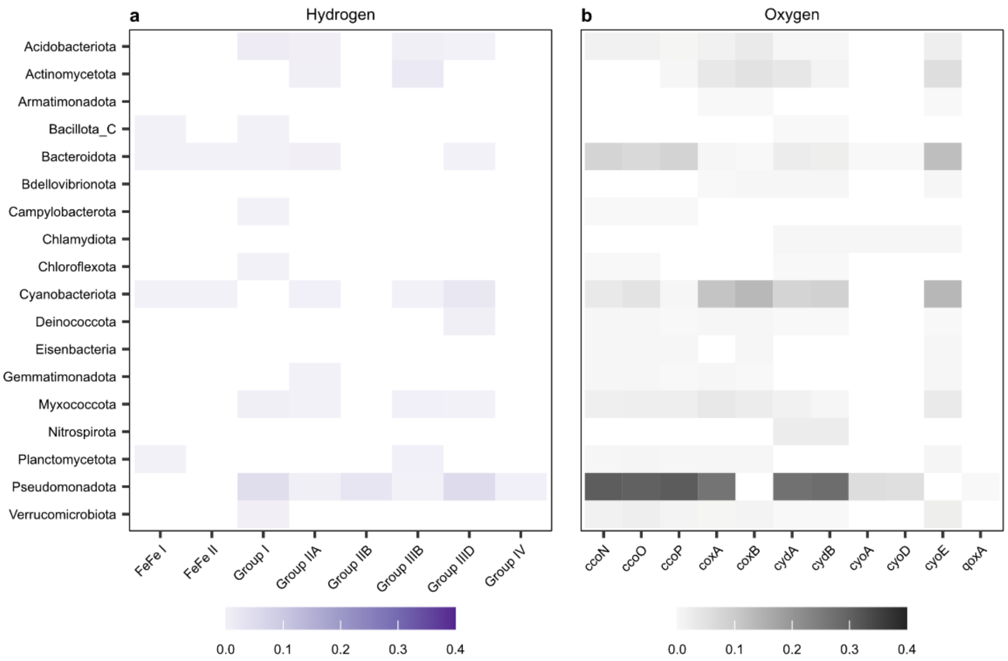

Supplementary Fig. 6. Hydrogen and oxygen cycling potential of bacterial MAGs. Heatmaps of (a) hydrogen, and (b) oxygen cycling genes where colour scales represent gene counts weighted by mean relative abundance and summed by bacterial phylum. Source data are provided as a Source Data file.

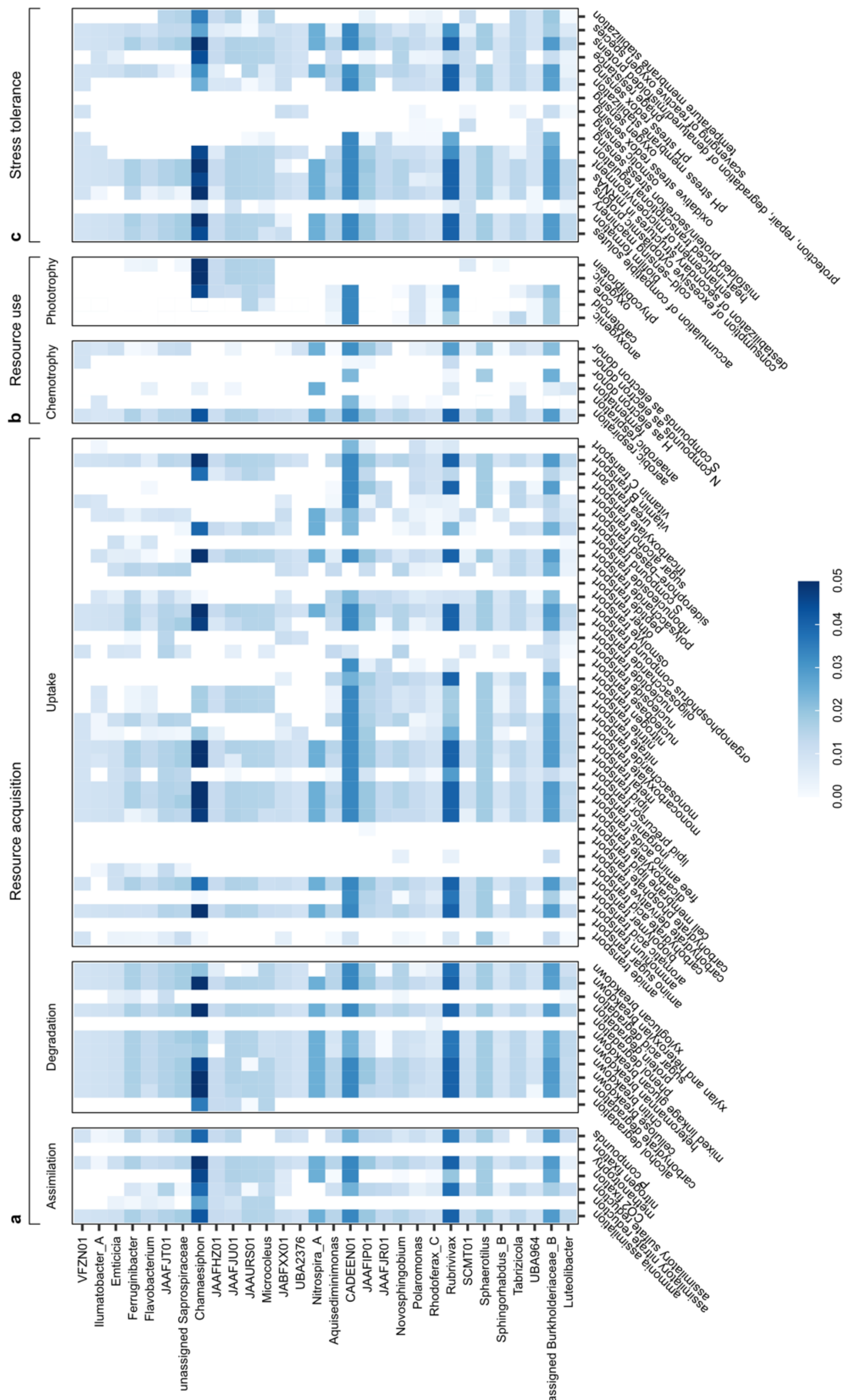

Supplementary Fig. 7. Functional genomic traits of bacterial MAGs presented at the genus level. Traits associated with (a) resource acquisition (substrate assimilation, degradation, and uptake), (b) resource use (chemotrophy and phototrophy), and (c) stress tolerance. The heatmap colour scale represents gene counts per category weighted by mean relative abundance and summed by bacterial genera. The 30 most abundant bacterial genera are shown, refer to Supplementary Fig. 5. for phylum assignments. Source data are provided as a Source Data file.

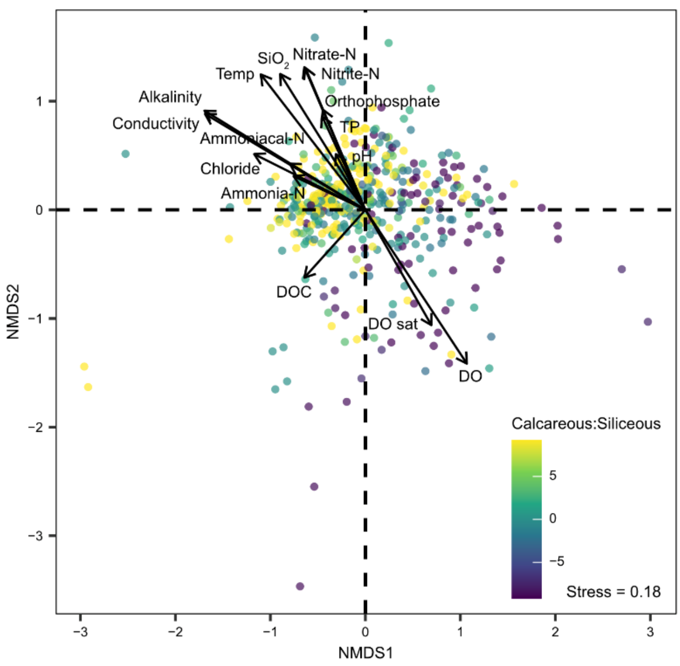

Supplementary Fig. 8. Beta-diversity of river biofilm bacterial communities and relationship with geology and water chemistry. Non-metric multidimensional scaling (NMDS) of a Bray-Curtis dissimilarity matrix based on beta diversity, where point colour indicates the log ratio of the dominant geological drivers: calcareous to siliceous geology (measured as the proportion of the upstream catchment represented by each geology type). Water chemistry variables are fitted to the ordination space, where vector length is proportional to the strength of the correlation. Source data are provided as a Source Data file.

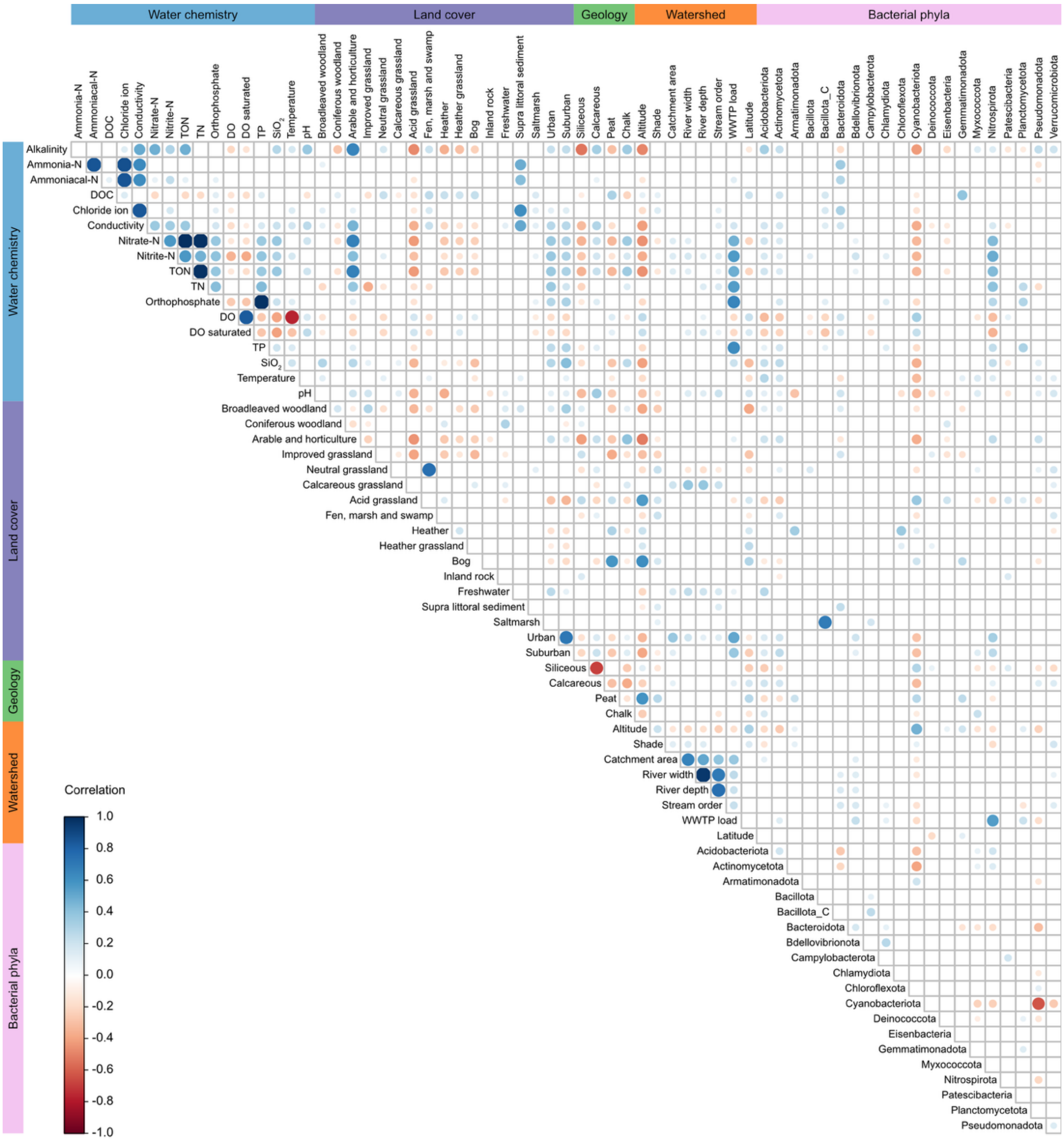

Supplementary Fig. 9. Pearson correlations between environmental variables and bacterial phyla. Heatmap scale indicates strength of correlation, with blue representing positive correlations and red representing negative correlations. Point size is proportional to the strength of correlation. Only significant ($p < 0.05$) correlations are shown. Source data are provided as a Source Data file.
