# Supplementary material for: National-scale biogeography and function of river and stream bacterial biofilm communities
Source: Nat Commun. 2025 Nov 26;16:10571. doi: 10.1038/s41467-025-65620-3 (PMC12657883; doi:10.1038/s41467-025-65620-3)
Supplement: Supplementary file 2 — Description of Additional Supplementary Files [file 41467_2025_65620_MOESM2_ESM.pdf]

Description of additional supplementary files

File name: Supplementary Data 1

Description: environmental_metadata.xlsx: environmental metadata associated with each sample, sheet 1: water chemistry, sheet 2: catchment land cover, sheet 3: catchment geology, sheet 4: watershed characteristics, sheet 5: data sources.

File name: Supplementary Data 2

Description: singlem_results.csv: proportion of archaea, bacteria, and eukaryotes in the pre-processed metagenomic reads.

File name: Supplementary Data 3

Description: MAG_metadata.xlsx: metadata associated with bacterial MAGs, sheet 1: coverage, sheet 2: CheckM2 statistics, sheet 3: GTDB-Tk output, sheet 4: Levins' index.

File name: Supplementary Data 4

Description: metabolism_and_functions.xlsx: presence of metabolic and functional traits identified in the bacterial MAGs, sheet 1: METABOLIC results, sheet 2: metabolisHMM results, sheet 3: microTrait results, sheet 4: biofilm-associated KO counts, sheet 5: biofilm associated-KO list.

File name: Supplementary Data 5

Description: varPart_and_correlations.xlsx: variance partitioning at the MAG level and correlation analysis, sheet 1: variance partitioning, sheet 2: Pearson correlations, sheet 3: NMDS envfit results.
