# Supplementary material for: National-scale biogeography and function of river and stream bacterial biofilm communities
Source: Nat Commun. 2025 Nov 26;16:10571. doi: 10.1038/s41467-025-65620-3 (PMC12657883; doi:10.1038/s41467-025-65620-3)
Supplement: Supplementary file 8 — Reporting summary [file 41467_2025_65620_MOESM8_ESM.pdf]

nature portfolio

Corresponding author(s): Amy Thorpe, Susheel Bhanu Busi, Daniel Read

Last updated by author(s): 03/10/2025

Reporting Summary

Nature Portfolio wishes to improve the reproducibility of the work that we publish. This form provides structure for consistency and transparency in reporting. For further information on Nature Portfolio policies, see our Editorial Policies and the Editorial Policy Checklist.

Statistics

For all statistical analyses, confirm that the following items are present in the figure legend, table legend, main text, or Methods section.

n/a	Confirmed	
☐	☒	The exact sample size (*n*) for each experimental group/condition, given as a discrete number and unit of measurement
☐	☒	A statement on whether measurements were taken from distinct samples or whether the same sample was measured repeatedly
☐	☒	The statistical test(s) used AND whether they are one- or two-sided<br>*Only common tests should be described solely by name; describe more complex techniques in the Methods section.*
☐	☒	A description of all covariates tested
☐	☒	A description of any assumptions or corrections, such as tests of normality and adjustment for multiple comparisons
☐	☒	A full description of the statistical parameters including central tendency (e.g. means) or other basic estimates (e.g. regression coefficient) AND variation (e.g. standard deviation) or associated estimates of uncertainty (e.g. confidence intervals)
☐	☒	For null hypothesis testing, the test statistic (e.g. *F*, *t*, *r*) with confidence intervals, effect sizes, degrees of freedom and *P* value noted<br>*Give P values as exact values whenever suitable.*
☒	☐	For Bayesian analysis, information on the choice of priors and Markov chain Monte Carlo settings
☐	☒	For hierarchical and complex designs, identification of the appropriate level for tests and full reporting of outcomes
☐	☒	Estimates of effect sizes (e.g. Cohen's *d*, Pearson's *r*), indicating how they were calculated

Our web collection on statistics for biologists contains articles on many of the points above.

Software and code

Policy information about availability of computer code

Data collection	Snakemake workflows used to process and analyse the metagenomic data are available at: https://github.com/amycthorpe/metag_analysis_EA and https://github.com/amycthorpe/EA_metag_post_analysis. R scripts for data analysis and visualisation are available at: https://github.com/amycthorpe/biofilm_MAG_analysis.
Data analysis	Software used: Snakemake v7.8.2, Trim Galore v0.6.5, singleM v0.16.0, MultiQC v1.17, Megahit v1.2.9, Prodigal v2.6.3, EggNOG-mapper v2.1.9, Kraken2 v2.1.2, Bracken v2.6.0, MetaBAT v2.15, MetaBinner v1.4.3, CONCOCT v1.1.0, CheckM2 v1.2.2, GTDBtk v2.3.2, microTrait v1.0.0, METABOLIC v4.0, metabolisHMM v2.22, MicrobeAnnotator v2.0.5, ggtree v3.12.0, MicroNiche v1.0.0, variancePartition v1.34.0, R v4.4.0

For manuscripts utilizing custom algorithms or software that are central to the research but not yet described in published literature, software must be made available to editors and reviewers. We strongly encourage code deposition in a community repository (e.g. GitHub). See the Nature Portfolio guidelines for submitting code & software for further information.

Data

Policy information about availability of data

All manuscripts must include a data availability statement. This statement should provide the following information, where applicable:

- Accession codes, unique identifiers, or web links for publicly available datasets
- A description of any restrictions on data availability
- For clinical datasets or third party data, please ensure that the statement adheres to our policy

The metagenomic data generated in this study have been deposited in the European Nucleotide Archive (ENA) at EMBL-EBI under accession number PRJEB85861. Sample accession codes and all the data generated, including the environmental metadata associated with each sample, MAG coverage, taxonomy, and CheckM2 statistics, outputs from METABOLIC, metabolisHMM, and microTrait, and the niche breadth index, variance partitioning, and correlation analysis results are available on Zenodo at: https://doi.org/10.5281/zenodo.14762144. Source data are provided with this paper.

Research involving human participants, their data, or biological material

Policy information about studies with human participants or human data. See also policy information about sex, gender (identity/presentation), and sexual orientation and race, ethnicity and racism.

Reporting on sex and gender	*Use the terms sex (biological attribute) and gender (shaped by social and cultural circumstances) carefully in order to avoid confusing both terms. Indicate if findings apply to only one sex or gender; describe whether sex and gender were considered in study design; whether sex and/or gender was determined based on self-reporting or assigned and methods used.* *Provide in the source data disaggregated sex and gender data, where this information has been collected, and if consent has been obtained for sharing of individual-level data; provide overall numbers in this Reporting Summary. Please state if this information has not been collected.* *Report sex- and gender-based analyses where performed, justify reasons for lack of sex- and gender-based analysis.*
Reporting on race, ethnicity, or other socially relevant groupings	*Please specify the socially constructed or socially relevant categorization variable(s) used in your manuscript and explain why they were used. Please note that such variables should not be used as proxies for other socially constructed/relevant variables (for example, race or ethnicity should not be used as a proxy for socioeconomic status).* *Provide clear definitions of the relevant terms used, how they were provided (by the participants/respondents, the researchers, or third parties), and the method(s) used to classify people into the different categories (e.g. self-report, census or administrative data, social media data, etc.)* *Please provide details about how you controlled for confounding variables in your analyses.*
Population characteristics	*Describe the covariate-relevant population characteristics of the human research participants (e.g. age, genotypic information, past and current diagnosis and treatment categories). If you filled out the behavioural & social sciences study design questions and have nothing to add here, write "See above."*
Recruitment	*Describe how participants were recruited. Outline any potential self-selection bias or other biases that may be present and how these are likely to impact results.*
Ethics oversight	*Identify the organization(s) that approved the study protocol.*

Note that full information on the approval of the study protocol must also be provided in the manuscript.

Field-specific reporting

Please select the one below that is the best fit for your research. If you are not sure, read the appropriate sections before making your selection.

☐ Life sciences ☐ Behavioural & social sciences ☒ Ecological, evolutionary & environmental sciences

For a reference copy of the document with all sections, see nature.com/documents/nr-reporting-summary-flat.pdf

Ecological, evolutionary & environmental sciences study design

All studies must disclose on these points even when the disclosure is negative.

Study description	Biofilm samples (n=450 samples) were collected from rivers across England (n=146 sites) for a national-scale study into biofilm microbial biogeography, composition, and function, and an investigation into how their communities are shaped by environmental drivers. DNA was extracted from each biofilm sample for shotgun metagenomic sequencing and metagenome assembled genomes (MAGs) were assembled. This was paired with detailed and high resolution environmental monitoring and catchment metadata.
Research sample	Samples were benthic river biofilms and the microbial community inhabiting the biofilms were characterised with metagenomic sequencing. Biofilms were chosen because they host taxonomically and functionally diverse microbial communities, are metabolically active, are found in freshwater environments globally, and are highly sensitive to environmental change. However, river biofilm microbial communities are poorly understood in comparison to river sediment or water column communities.
Sampling strategy	Biofilm samples were collected across 146 sites representative of England's diverse river network. These sites encompassed a wide

	range of land cover types, geologies, and water chemistry parameters (Supplementary Data 1). Samples were collected in multiple seasons and across multiple years to capture seasonal variation and ensure samples were representative of river biofilm communities in England. The sampling sites were selected using a randomised, spatially balanced design to ensure representative and unbiased spatial coverage across England (Generalized Random Tessellation Stratified sampling, https://doi.org/10.1016/j.proenv.2015.07.108).
Data collection	Samples and environmental data were collected from each site by the Environment Agency as part of their routine monitoring. Water temperature measurements were made using a probe, for all water chemistry measurements 1L samples were taken and shipped to the laboratory for analysis. Up to 5 measurements of each water chemistry variable were taken at each site over a 3-month period prior to biofilm sampling and this data is available at: https://environment.data.gov.uk/water-quality/view/landing.
Timing and spatial scale	Samples were collected as part of routine monitoring across England spanning a latitudinal gradient of 645 km. Samples were collected in 2021, 2022 and 2023 between March and November.
Data exclusions	No samples were excluded and all DNA extracts sequenced successfully.
Reproducibility	All protocols are provided for reproducibility and transparency. This includes detailed descriptions of sampling, sequencing, data processing and subsequent analysis provided in the methods, in addition to the provision of all code on GitHub (https://github.com/amycthorpe/metag_analysis_EA, https://github.com/amycthorpe/EA_metag_post_analysis, https://github.com/amycthorpe/biofilm_MAG_analysis).
Randomization	Biofilms were sampled from five stones randomly selected from the river at each site.
Blinding	Samples were extracted and sequenced in a randomised order and sample IDs did not reveal any information about site location.

Did the study involve field work? ☒ Yes ☐ No

Field work, collection and transport

Field conditions	Environmental conditions measured at each site are provided in Supplementary Data 1 and on Zenodo at: https://doi.org/10.5281/zenodo.14864644.
Location	Rivers and streams across England with a latitudinal gradient spanning 645 km. See Fig. 1A for sampling site locations.
Access & import/export	All samples were collected in a responsible manner with landowners permission where required.
Disturbance	All sampling was done in a manner to minimise disturbance to the local environment, when required samplers would travel by foot to the sampling site from the nearest available parking.

Reporting for specific materials, systems and methods

We require information from authors about some types of materials, experimental systems and methods used in many studies. Here, indicate whether each material, system or method listed is relevant to your study. If you are not sure if a list item applies to your research, read the appropriate section before selecting a response.

Materials & experimental systems

n/a	Involved in the study
☒	☐ Antibodies
☒	☐ Eukaryotic cell lines
☒	☐ Palaeontology and archaeology
☒	☐ Animals and other organisms
☒	☐ Clinical data
☒	☐ Dual use research of concern
☒	☐ Plants

Methods

n/a	Involved in the study
☒	☐ ChIP-seq
☒	☐ Flow cytometry
☒	☐ MRI-based neuroimaging

Plants

Seed stocks	*Report on the source of all seed stocks or other plant material used. If applicable, state the seed stock centre and catalogue number. If plant specimens were collected from the field, describe the collection location, date and sampling procedures.*
Novel plant genotypes	*Describe the methods by which all novel plant genotypes were produced. This includes those generated by transgenic approaches, gene editing, chemical/radiation-based mutagenesis and hybridization. For transgenic lines, describe the transformation method, the number of independent lines analyzed and the generation upon which experiments were performed. For gene-edited lines, describe the editor used, the endogenous sequence targeted for editing, the targeting guide RNA sequence (if applicable) and how the editor was applied.*
Authentication	*Describe any authentication procedures for each seed stock used or novel genotype generated. Describe any experiments used to assess the effect of a mutation and, where applicable, how potential secondary effects (e.g. second site T-DNA insertions, mosiacism, off-target gene editing) were examined.*
